# Advances in Mineral Processing, Waste Recycling and Extractive Metallurgy

**DOI:** 10.3390/ma17010133

**Published:** 2023-12-27

**Authors:** Dmitry Valeev, Alex Kondratiev, Jinhe Pan

**Affiliations:** 1 Laboratory of Sorption Methods, Vernadsky Institute of Geochemistry and Analytical Chemistry, The Russian Academy of Sciences, 119991 Moscow, Russia; 2 Laboratory of Chemical Thermodynamics, Department of Physical Chemistry, Faculty of Chemistry, Lomonosov Moscow State University, Leninskie Gory, 1, 119991 Moscow, Russia; al.v.kondratiev@gmail.com; 3 Key Laboratory of Coal Processing and Efficient Utilization, Ministry of Education, School of Chemical Engineering and Technology, China University of Mining and Technology, Xuzhou 221116, China; jiinhepan@cumt.edu.cn

## 1. Introduction and Scope

**Figure 1:**
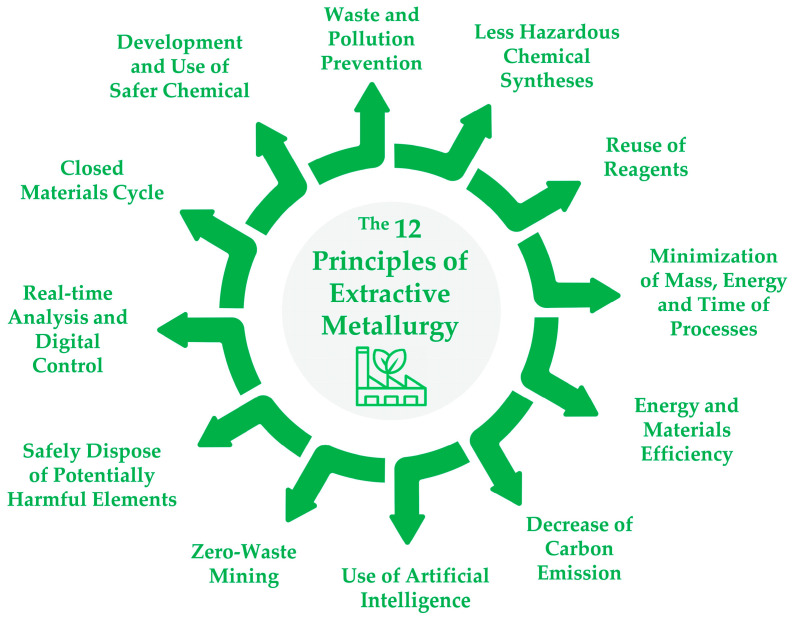
The conceptual flowsheet of circular extractive metallurgy (some principles were adapted from green chemistry engineering [1] and hydrometallurgy [2]).

The constant growth of the world economy and industry stimulates an increasing production of ferrous and non-ferrous metals, while the depletion of natural resources leads to demands for the development of new technologies for the processing of low-grade ores and the deep recycling of metallurgical and other anthropogenic wastes. These new technologies should follow the twelve principles of extractive metallurgy (Figure 1) and will play an important role in creating a sustainable future for mankind.

This Special Issue of *Materials* is devoted to the most relevant research relating the pyro- and hydrometallurgy methods for the extraction and recycling of ferrous and non-ferrous metals, including studies on the chemical/phase composition and physical properties of relevant products (concentrates, slags, metals, oxides, solutions, salts, etc.). The Guest Editors of the Special Issue were Dr. Alex Kondratiev, Dr. Dmitry Valeev and Dr. Jinhe Pan, and have purposed it to collate submissions discussing the following topics: platinum-group metals, solvent extraction, noble metal recovery, acid leaching, thermal infrared hyperspectral technology, potassium salts, quantitative analysis of mixed minerals and finite mineral phases, stockpiled discard coal, waste polyethylene terephthalate, co-carbonization, metallurgical coke, thermodynamic stability constants, carbonate complexes, thermodynamics of the formation of rare earth elements, chlorite, carboxymethyl cellulose, froth entrainment, flocculation, agglomeration, pellet fines, organic binders, briquettes, reduction, green steel, boehmite, atmospheric leaching, hematite reduction, red mud valorization, Mössbauer spectroscopy, activated carbon, adsorption, gold, heap leaching, germanium and lignite, among others. The suggested application areas include ferrous and non-ferrous metallurgy. Ten research articles from Russia, China, Poland, South Africa, Sweden and Mexico were selected, peer-reviewed and accepted for publication.

## 2. Contributions

Pianowska et al. [3] submitted an article entitled “Solvent Extraction as a Method of Recovery and Separation of Platinum Group Metals”. Platinum-group metals (PGMs) comprise six metals with high market value and key importance to many industrial sectors. Due to their low prevalence in the Earth’s crust and high demand, these metals have been recognized as critical materials for many years. Along with economic development, the natural resources of platinum-group metals are gradually being depleted, accompanied by the need to recover PGMs from secondary sources. The solutions resulting from the processing of such materials yield products characterized by a high content of impurities and low content of precious metals. As such, in order to obtain pure metals, it is extremely important to choose an effective, selective method for the recovery and separation of platinum-group metals. This review focuses on the most important aspects of PGM characteristics, including their properties and occurrence, the processing of natural and secondary raw materials and the role of liquid–liquid extraction in the selective separation of metals from this group, not only at the laboratory scale but, above all, at the industrial scale. In addition, this study collects information on the most commonly used commercially available extractants based on current reports within the scientific literature.

Shoppert et al. [4] contributed an article entitled “Rare-Earth Elements Extraction from Low-Alkali Desilicated Coal Fly Ash by (NH4)_2_SO_4_ + H_2_SO_4_”. Coal fly ash (CFA) obtained from pulverized coal furnaces is a highly refractory waste that can be used for alumina and rare earth element (REE) extraction. REEs in this type of CFA are associated with a mullite and amorphous glassy mass that forms a core-shell structure. In this research, it was shown that the complete dissolution of amorphous aluminosilicates from the mullite surface with the formation of the low-alkali mullite concentrate prior to sulfuric acid leaching with the addition of (NH_4_)_2_SO_4_ helps accelerate the extraction of REEs. The extraction degree of Sc and other REEs reaches 70–80% after 5 h of leaching at 110 °C and an acid concentration of 5 M versus less than 20% for the raw CFA under the same conditions. To study the leaching kinetics of the process, the effects of temperature (90–110 °C), liquid-to-solid ratio (5–10), and leaching time (15–120 min) on the degrees of Al and rare earth element (REE) extraction were evaluated. After 120 min of leaching at 110 °C and L/S ratio = 10, the extraction of Al was found to be lower than 30%. At the same time, total REE (TREE) and Fe extraction was greater than 60%, which indicates that part of the TREE was transferred into the acid-soluble phase. After leaching, the residues were studied via laser diffraction (LD), X-ray diffraction (XRD), X-ray fluorescence (XRF) and scanning electron microscopy (SEM-EDS) to evaluate the leaching mechanism and solubility of Al- and Fe-containing minerals such as mullite, hematite and amorphous aluminosilicate.

Qi et al. [5] provided an article entitled “Quantitative Analysis of Mixed Minerals with Finite Phase Using Thermal Infrared Hyperspectral Technology”. It is crucial but challenging to detect intermediate or end products promptly. Traditional chemical detection methods are time-consuming and cannot detect mineral phase content. Thermal infrared hyperspectral (TIH) technology is an effective means of real-time imaging and can precisely capture the emissivity characteristics of objects. This study introduces TIH to estimate the content of potassium salts, with a model based on competitive adaptive reweighted sampling (CARS) and partial least-squares regression (PLSR). The model takes the emissivity spectrum of potassium salt into account and accurately predicts the content of mixing potassium (MP), a mineral mixture produced in Lop Nur, Xinjiang. The main mineral content in MP was measured using a mineral liberation analyzer (MLA), including picromerite, potassium chloride, magnesium sulfate and less sodium chloride. The 129 configured MP samples were divided into calibration (97 samples) and prediction (32 samples) sets. The CARS-PLSR method achieved good prediction results for MP mineral content (picromerite: correlation coefficient of correction set ($R_{p}^{2}$) = 0.943, predicted root mean square error (RMSEP) = 2.72%, relative predictive deviation (RPD) = 4.24; potassium chloride: $R_{p}^{2}$ = 0.948, RMSEP = 2.86%, RPD = 4.42). Experimental results convey that TIH technology can effectively identify the emissivity characteristics of MP minerals, facilitating the quantitative detection of MP mineral content.

Bambalaza et al. [6] submitted an article entitled “Co-Carbonization of Discard Coal with Waste Polyethylene Terephthalate towards the Preparation of Metallurgical Coke”. Waste plastics such as polyethylene terephthalate (w-PET) and stockpiled discard coal (d-coal) pose a global environmental threat, as they are disposed of in large quantities as solid waste into landfills and are particularly hazardous due to the spontaneous combustion of d-coal that produces greenhouse gases (GHGs) and the non-biodegradability of w-PET plastic products. This study reports on the development of a composite material, prepared from w-PET and d-coal, with physical and chemical properties similar to those of metallurgical coke. The w-PET/d-coal composite was synthesized via a co-carbonization process at 700 °C under a constant flow of nitrogen gas. Proximate analysis results showed that a carbonized w-PET/d-coal composite could attain up to 35% improvement in fixed carbon content compared to its d-coal counterpart, such that an initial fixed carbon content of 14–75% in carbonized discard coal could be improved to 49–86% in carbonized w-PET/d-coal composites. The results clearly demonstrate the role of d-coal ash on the degree of the thermo-catalytic conversion of w-PET to solid carbon, showing that the yield of carbon derived from w-PET (i.e., c-PET) was proportional to the ash content of d-coal. Furthermore, the chemical and physical characterization of the composition and structure of the c-PET/d-coal composite showed evidence of mainly graphitized carbon and a post-carbonization caking ability, similar to that of metallurgical coke. The results obtained in this study show potential for the use of waste raw materials, w-PET and d-coal in the development of an eco-friendly reductant with comparable chemical and physical properties to metallurgical coke.

Litvinova et al. [7] contributed an article entitled “Complex Formation of Rare-Earth Elements in Carbonate–Alkaline Media”. Rare earth metals are critical components of many industries. The extraction of rare earth metals from mineral raw materials presents many problems, both of technological and theoretical nature. The use of man-made sources imposes strict requirements on the process. Thermodynamic and kinetic data that could describe the most detailed technological water–salt leaching and precipitation systems are insufficient. The study addresses the problem of a small amount of data on the formation and equilibrium of carbonate–alkali systems of rare earth metals. The isotherms of solubility of sparingly soluble carbonates with the formation of carbonate complexes are presented to evaluate equilibrium constants logK at zero ionic strength for Nd—11.3, Sm—8.6, Gd—8.0 and Ho—7.3. To accurately predict the system under consideration, a mathematical model was developed which allows for calculating the water–salt composition. The initial data for calculation are concentration constants of stability of lanthanide complexes. This work will contribute to deepening knowledge about rare earth element extraction problems and will serve as a reference for studying the thermodynamics of water–salt systems.

Chen et al. [8] provided an article entitled “Effect of the Molecular Weight of Carboxymethyl Cellulose on the Flotation of Chlorite”. The present study aimed to investigate the influence mechanism of carboxymethyl cellulose (CMC) on the flotation of fine chlorite. To this end, a series of flotation tests, sedimentation tests and microscope analyses were conducted. Flotation tests revealed an inverse relationship between particle size and the recovery of chlorite, indicating that finer particles exhibited higher recovery rates. Moreover, it was observed that the recovery of fine chlorite was significantly associated with the water recovery (proportion of water entering the floated product to the weight of water in the initial flotation suspension) and a variety of froth types. Based on these findings, it can be inferred that froth entrainment may constitute a crucial component of the recovery mechanism underlying fine chlorite. Thus, reducing froth entrainment (the phenomenon of hydrophilic minerals entering floated products through foam water) is the key to depress chlorite flotation. Flotation tests indicate that fine chlorite recovered into froth products can be depressed effectively by CMC with a high molecular weight. The results of sedimentation tests and microscope analyses in the presence of CMC prove that CMC with a high molecular weight generates flocculation on fine chlorite particles, while CMC with a low molecular weight does not. It is suggested that the depression of chlorite flotation may be attributed to the reduction in the entrainment resulting from the flocculation induced by CMC.

Manu et al. [9] submitted an article entitled “Maximizing the Recycling of Iron Ore Pellets Fines Using Innovative Organic Binders”. This research work focuses on the practicality of using organic binders for the briquetting of pellet fines. The developed briquettes were evaluated in terms of mechanical strength and reduction behavior with hydrogen. A hydraulic compression testing machine and thermogravimetric analysis were incorporated into this work to investigate the mechanical strength and reduction behavior of the produced briquettes. Six organic binders, namely Kempel, lignin, starch, lignosulfonate, Alcotac CB6 and Alcotac FE14, in addition to sodium silicate, were tested for the briquetting of pellet fines. The highest mechanical strength was achieved using sodium silicate, Kempel, CB6 and lignosulfonate. The best combination of binder to attain the required mechanical strength even after 100% reduction was found to be a combination of 1.5 wt.% of organic binder (either CB6 or Kempel) with 0.5 wt.% of inorganic binder (sodium silicate). Upscaling using an extruder produced propitious results regarding the reduction behavior, as the produced briquettes were highly porous and attained pre-requisite mechanical strength.

Shoppert et al. [10] contributed an article entitled “Low-Temperature Treatment of Boehmitic Bauxite Using the Bayer Reductive Method with the Formation of High-Iron Magnetite Concentrate”. The Bayer process is the main method of alumina production worldwide. The use of low-quality bauxites for alumina production results in the formation of a significant amount of technogenic waste—bauxite residue (BR). The Bayer reductive method is one possible way to eliminate BR stockpiling, but it requires high-pressure leaching at temperatures higher than 220 °C. In this research, the possibility of boehmitic bauxite atmospheric pressure leaching at both the first and second stages or high-pressure leaching at the second stage with the simultaneous reduction of hematite to magnetite was investigated. Bauxite and solid residue after NaOH leaching were characterized using XRD, SEM-EDS and Mössbauer spectroscopy methods. The first stage of leaching under atmospheric pressure with the addition of Fe(II) species in a strong alkali solution (330–400 g L^−1^ Na_2_O) resulted in a partial reduction in iron minerals and an extraction of more than 60% of Si and 5–25% of Al (depending on caustic modulus of solution) after 1 h. The obtained desilicated bauxite was subjected to atmospheric leaching at 120 °C in a strong alkali solution (350 g L^−1^) or high-pressure leaching at 160–220 °C using the Bayer process mother liquor in order to obtain a concentrate with a magnetite content higher than 83 wt. %.

Martínez-Peñuñuriet al. [11] provided an article entitled “Thermodynamic and Kinetic Aspects of Gold Adsorption in Micrometric Activated Carbon and the Impact of Their Loss in Adsorption, Desorption, and Reactivation Plants”. The production and loss of fine particles of activated carbon (AC) loaded with gold in the adsorption processes is a worldwide problem, mainly due to its increasing adsorption capacity with respect to the decrease in particle size, which becomes relevant to determine the thermodynamic and kinetic activity of the gold adsorption and the economic impact of its loss, with the escape toward the later stages of the system of adsorption, desorption and reactivation (ADR) plants of AC. Through the adsorption of gold in a synthetic medium with sodium cyanide concentration, using different particle sizes, AC weights and adsorption times, data were generated for analysis using three different isotherm models, resulting in a better tendency for the Freundlich isotherm, from which thermodynamic parameters of ΔG equal to −2.022 kcal/mol, ΔH equal to −16.710 kcal/mol and ΔS equal to −0.049 kcal/molK were obtained, which shows that it is a spontaneous, exothermic process with a lower degree of disorder. The kinetic analysis was performed with two different models, from which the pseudo-second-order model was used due to a better tendency, and displayed a behavior that leaves the interpretation of the increase in adsorption with the decrease in the AC particle size open, but demonstrated the importance of recovering these particles in relation to their gold concentration and the economic impact from their poor recovery, which, for this case study, amounted to ~0.3 million dollars per year.

Yang et al. [12] contributed an article entitled “Extraction of Germanium from Low-Grade Germanium-Bearing Lignite by Reductive Volatilization”. Germanium (Ge), an important strategic metal, is widely used in many modern technology fields, such as optical fiber and thermal solar cells. In this study, the volatilization behavior of Ge from low-grade germanium-bearing lignite was investigated in detail via reductive volatilization. The results indicated that the temperature and air flow rate in the semi-closed roasting system played a significant role in the process. The optimal volitation efficiency of Ge reached 98% at 1100 °C for 2 h with an air flow rate of 0.7 L/min in a maffle furnace, respectively. Under optimal conditions, the contents of Ge lowered to 30 ppm in the roasting residue. Analysis of the enriched ash yielded 71,600 ppm for Ge. Chemical phase analysis of the Ge in the enrichment ash showed that soluble Ge accounted for 82.18% of the total Ge, which could be viewed as an excellent material for Ge extraction via chlorinated distillation.

## 3. Summary and Outlook

This Special Issue of *Materials* was well supported by numerous submissions, and the final publication consists of ten high-quality peer-reviewed articles. It is anticipated that due to this success, a new Special Issue (“Advances in Mineral Processing, Waste Recycling and Extractive Metallurgy (Second Edition)”, website: https://www.mdpi.com/journal/materials/special_issues/X3G3X41BTX accessed on 26 December 2023), will be commissioned as a follow-up to accept global contributions relating ferrous and non-ferrous metallurgy.
